# Cross-sectional study on sensitization to mite and cockroach allergen components in allergy patients in the Central European region

**DOI:** 10.1186/s13601-018-0207-x

**Published:** 2018-06-04

**Authors:** Petr Panzner, Martina Vachová, Tomáš Vlas, Petra Vítovcová, Petra Brodská, Marek Malý

**Affiliations:** 1 Department of Immunology and Allergology, Faculty of Medicine in Pilsen, Charles University, Pilsen, Czech Republic; 2 Department of Immunology and Allergology, Faculty Hospital in Pilsen, Pilsen, Czech Republic; 3 Department of Dermatovenerology, Faculty Hospital in Pilsen, Pilsen, Czech Republic; 4 The National Institute of Public Health, Prague, Czech Republic

## Abstract

**Background:**

The major sources of allergens in the indoor air include house dust mites, dander derived from domestic animals and rodents, cockroach, and several fungi. Mites are the main cause of allergies in some countries with a warmer climate, but the epidemiological significance of mite and cockroach allergens in Central Europe has not been established yet.

**Methods:**

We assessed sensitization profiles of allergy patients in a Central European region in regard to sensitization to mites and cockroach. We used molecular diagnosis by means of the microarray ISAC, and we investigated 1766 patients with clinical suspicion to an allergic disorder. 1255 of them were positive to at least one allergen component, and this group was subjected to statistical analysis.

**Results:**

The sensitization to at least one mite-specific molecule (Der p 1, 2, Der f 1, 2) was observed relatively frequently in 32.7% of patients. Specific IgE to mite group 2 molecules is almost fully cross-reactive. Group 1 allergens are also cross-reactive, but in some patients, a species-specific response was observed. Relatively high rate of sensitization both to group 1 and 2 allergens in our patients indicates the greater role of co-sensitizations. Isolated sensitizations to molecules derived from glyciphagid mites Lep d 2 and/or Blo t 5 without sensitization to other mite-derived molecules were observed only exceptionally (in 0.6% of cases). True sensitization to at least one cockroach-specific molecule (Bla g 1, 2, 5) was very rare (in 0.6% of cases), and nearly all of them were co-sensitizations with other noncockroach-derived molecules. Sensitization to an inhaled tropomyosin was observed rarely in 2.2% of patients (Der p 10 in 1.9% and Bla g 7 in 1.5%). Co-sensitization of inhaled tropomyosins with the respective mite- or cockroach-specific molecules was observed only in the minority of patients suggesting the different route of sensitization being more frequent.

**Conclusions:**

The majority of patients are co-sensitized to several molecules of the respective allergen source. The knowledge of this molecular spectrum of sensitization is important for optimal diagnosis and treatment in respect to allergen content in mite extracts used for diagnostic and therapeutic purposes. In regard to the sensitization patterns of Central European patients, it is necessary to point out the importance of quantifying at least three major mite components Der f 1, Der p 1 and Der f 2 (or Der p 2).

## Background

Immediate hypersensitivity to indoor allergens is a risk factor for asthma, and allergic rhinitis and sensitization to these allergens may play a role in atopic dermatitis as well. The major sources of allergens in the indoor air include house dust mite (HDM), dander derived from domestic animals and rodents, cockroach, and several fungi. Given that most persons in Western societies spend more than 90% of their lives in indoor environments, it is not surprising that indoor allergens play an important role in allergic sensitization and symptoms. Sensitization to distinct molecules may represent higher risk for asthma, or atopic dermatitis [1, 2] and several studies suggest that sensitization to multiple molecules (“molecular spreading”) is associated with a higher probability of more severe symptoms of allergy [2–4].

HDM is the main cause of allergies in some countries with a warmer climate [5]; in Central Europe, the sensitization rates to mites, some animals (especially cats and dogs) and molds (especially Alternaria) immediately follow the sensitization rates to pollens [6, 7]. Cockroach allergy is an important cause of asthma in several regions of America and Asia [8], its significance in Central Europe has not been established yet.

While the diagnosis of immunoglobulin E (IgE)-mediated inhalant allergy is primarily based on clinical history and sensitization, that is demonstrated via skin prick testing and measurement of serum allergen-specific IgE; this methodology has its limitations. In vitro and in vivo allergy testing are often based on, the insufficiency of standardized allergen extracts owing to the natural variability of the allergen source, or manufacturing procedure, can differ regarding their allergenic content. This issue was already confirmed also for HDM allergens [9–11]. An even more important disadvantage of allergenic extracts is that they are incapable of differentiating between primary sensitization and immunological cross-reactivity in multiple sensitizations which are observed in many patients. Nonetheless, natural allergenic extracts were the cornerstone of inhalant allergy diagnosis until several years ago, when the molecular diagnosis was made possible by advances in molecular biology which lead to the development of a large spectrum of purified natural and recombinant allergenic molecules. Such presently routinely available reagents enable the use of the diagnostic approach commonly known as a component-resolved diagnosis of allergy, and now allow the systematic study of the principal allergens and cross-reactivity processes involved in allergic sensitization.

The introduction of microarrays with a much larger number of purified or recombinant molecules constituted a further development in the diagnosis of allergic diseases. Such microarrays now represent powerful tools in the screening of serum IgE-reactivity, and they allow the definition of sensitization profiles. Identification of sensitizations and co-sensitizations to species-specific and cross-reacting allergen components may be especially important in decisions concerning allergen-specific immunotherapy.

This study aimed to assess the usefulness of molecular diagnosis using a microarray in the description of sensitization profiles in subjects showing a sensitization to HDM and cockroach living in the Central European region, with a special focus on discriminating between cross-reactivities and multiple sensitizations to different allergens. Although not much data on the diagnostic accuracy of the microarray ImmunoCAP ISAC in HDM and cockroach allergy is available, we decided to use this approach because of the possibility it provides to analyze a wide spectrum of component sensitizations. Furthermore, some studies have shown similar performances for component-based microarray ISAC and whole-allergen CAP system detection [12, 13]. Nonetheless, it is necessary to bear in mind the possible different sensitivities to individual molecules in the used assay.

## Methods

This cross-sectional observational study was conducted according to the STROBE recommendations [14] to the extent which may apply to this study design. We retrospectively analyzed data from 1766 patients who had been examined in the years 2011–2014 based on suspicion of allergy at the outpatient service of the Department of Immunology and Allergology of the University Hospital in Pilsen; the patients came from the western part of the Czech Republic. One thousand two hundred fifty-five patients positive to at least one allergen component were subjected to further detailed analysis. This test group of 1255 sensitized patients had at least one of the following diagnoses: chronic rhinitis (73%), bronchial asthma (41%), atopic dermatitis (34%), urticaria or edema (19%), and/or anaphylaxis (11%). Patient ages ranged from 1 to 68 years, with a mean age of 29 years. The sex ratio was 45.3% men to 54.7% women.

The detection of specific IgE to multiple allergen components was performed using the 112 component ImmunoCAP ISAC allergen microarray immunoassay (Thermo Fisher Scientific, Uppsala, Sweden). Briefly, microarray reaction sites were incubated with 20 μl undiluted patient serum for 2 h to capture allergen-specific IgE antibodies by their corresponding allergen. Subsequently, the microarray slides were rinsed and washed to remove unbound sIgE. After drying, complexes of allergen-bound sIgE were stained with a secondary, fluorescence-labeled anti-human IgE for 1 h at room temperature while protected from light. After a second rinsing and washing procedure, the obtained fluorescence signals were scanned using a laser scanner (LuxScan 10K; CapitalBio, Beijing, China). Analysis of the corresponding digitized microarray images was performed using ImmunoCAP ISAC software, and image information was transformed into numerical data according to a reference serum of known IgE content. Results were expressed as ISAC standardized units (ISU), and values greater than or equal to 0,3 ISU/l were taken as positive.

The analysis was focused on inhalant mite- and cockroach-derived specific allergen components and potentially cross-reactive components which are included in the ISAC system. Specific allergy markers are represented by the group 1 (nDer p 1, nDer f 1), group 2 (rDer p 2, rDer f 2, rLep d 2) and group 5/21 (rBlo t 5) allergens for mites, and molecule rBla g 1, aspartic protease rBla g 2 and glutathione-S-transferase rBla g 5 for cockroach. Finally, panallergens like mite- and cockroach-derived tropomyosins (rDer p 10 and nBla g 7) were also included in the analysis and related to sensitizations to other tropomyosins (nPen m 1 and rAni s 3).

## Results

The results of the analysis describing the HDM and cockroach sensitization patterns in the group of 1255 patients sensitized to at least one allergen component in our region are listed below. All percentages were calculated using the whole group of 1255 patients.

### Mites

The sensitization to at least one mite-specific molecule (Der p 1, 2, Der f 1, 2) was observed in 32.7% of patients. Isolated sensitizations to molecules derived from glyciphagid mites Lep d 2 and/or Blo t 5 without sensitization to other mite-derived molecules were observed only in 0.6% of cases.

### Cockroach

True sensitization to at least one cockroach-specific molecule (Bla g 1, 2, 5) was very rare (in 0.6% of cases), and nearly all of them were co-sensitizations with other non-cockroach-derived molecules, including mite-derived molecules in half of the patients (4 cases).

### Tropomyosins

Sensitization to an inhaled mite- and cockroach-derived tropomyosin was observed in 2.2% of patients (Der p 10 in 1.9% and Bla g 7 in 1.5%).

Co-sensitization of Der p 10 with other mite-derived molecules was observed in 0.7% of patients. Co-sensitization of Bla g 7 with other cockroach-specific molecules was exceptional (in 0.1%), without co-sensitization to Der p 10 in all cases.

In patients sensitized to Der p 10 and not to Bla g 7 (0.6%) no sensitization to other tropomyosins (Pen m 1, Ani s 3) was observed in nearly all of the cases (0.5%), and sensitization to other mite-derived molecules was observed only in 0.2% of cases. In patients sensitized both to Der p 10 and Bla g 7 (1.3%), sensitization to other tropomyosins was observed in all cases, and sensitization to other mite-derived molecules was observed in 0.5% of cases. Monosensitization (in the frame of tropomyosins) to Bla g 7 was only exceptional.

**Fig. 1:**
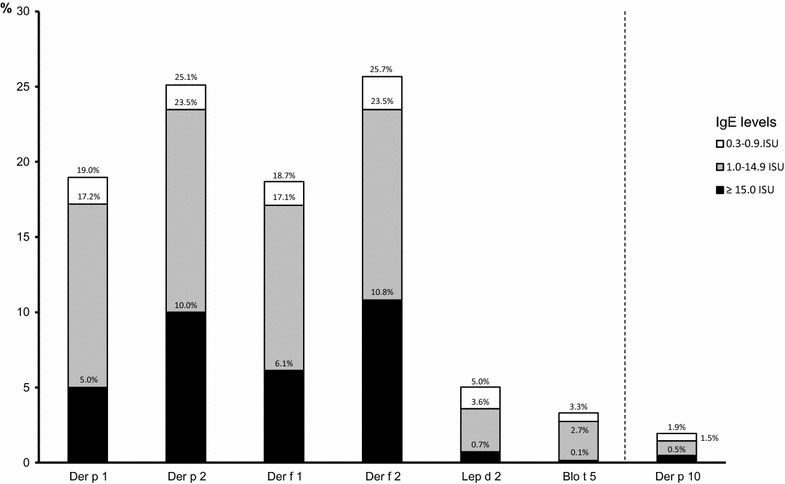
Sensitization rates to mite-derived molecules

**Fig. 2:**
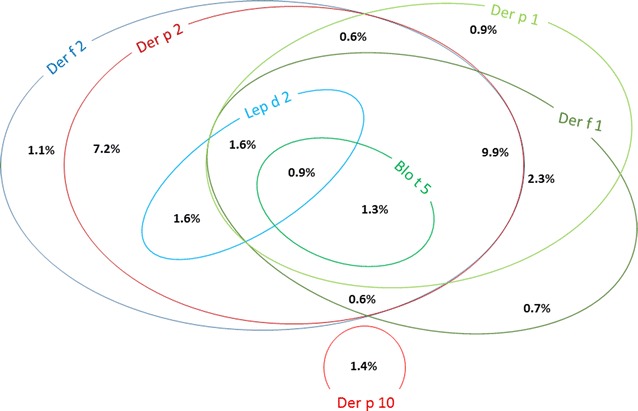
Venn diagram depicting mono- and co-sensitizations to mite-derived molecules. Mono- and co-sensitizations with a frequency of less than 0.6% are not shown

**Fig. 3:**
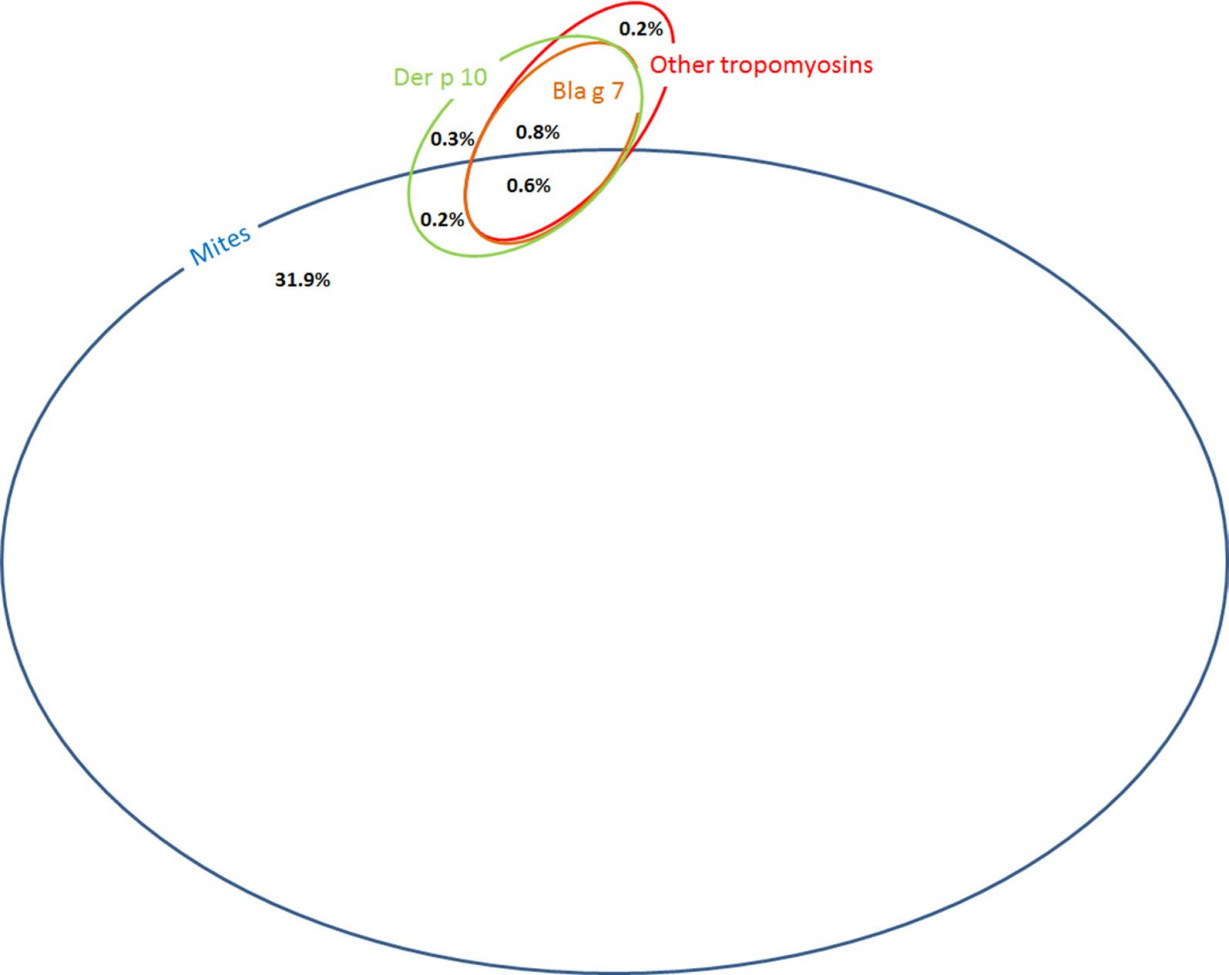
Venn diagram depicting mono- and co-sensitizations to tropomyosins in relation to sensitization to mites (= sensitization to at least one mite-specific molecule). Mono- and co-sensitizations with a frequency of less than 0.2% are not shown

**Fig. 4:**
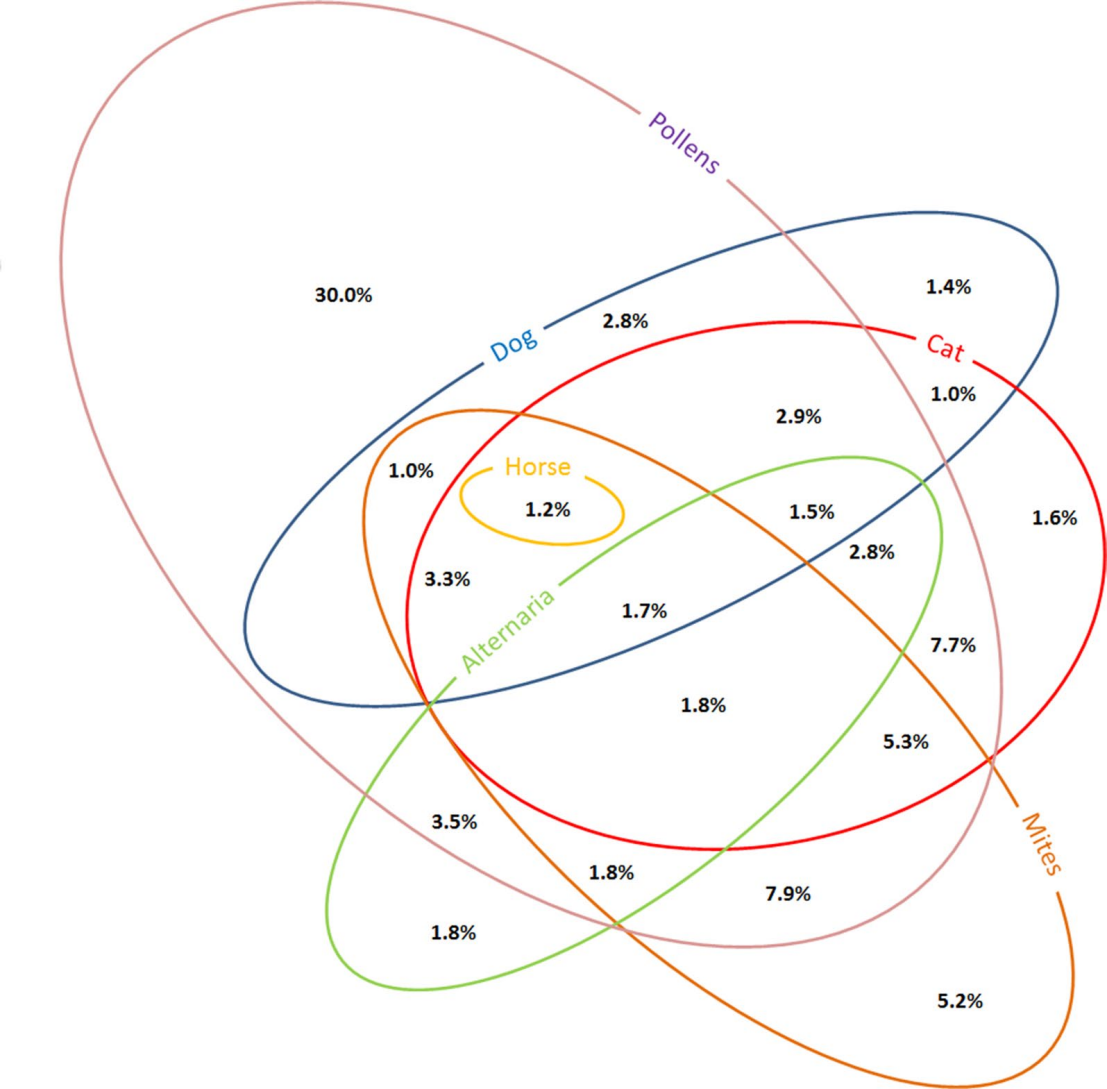
Venn diagram depicting mono- and co-sensitizations to different groups of molecules—mites and other inhalant allergens. Sensitization to a group is defined as sensitization to at least one of the respective species-specific component: mites (Der p 1, Der p 2, Der f 1, Der f 2), cat (Fel d 1, Fel d 4), dog (Can f 1, Can f 2, Can f 5), horse (Equ c 1), Alternaria (Alt a 1, Alt a 6), pollens (Phl p 1, Phl p 2, Phl p 4, Phl p 5, Phl p 6, Phl p 11, Cyn d 1, Bet v 1, Aln g 1, Cor a 1, Cup a 1, Cry j 1, Ole e 1, Ole e 9, Pla a 2, Pla l 1, Art v 1, Che a 1). Mono- and co-sensitizations with a frequency of less than 1.0% are not shown

The frequency of sensitization to individual mite-derived molecules, and their co-sensitizations is shown in Figs. 1 and 2. Sensitizations and co-sensitizations in the frame of tropomyosins and mites are shown in Fig. 3. The sensitization to mite-specific molecules in the context of sensitizations to molecules specific for other inhalant allergens is shown in Fig. 4.

## Discussion

The observed sensitization rate to perennial inhalant-derived molecules in our group of patients is lower than the sensitization rate to pollen-derived components [6]. However, the relatively high sensitization rate to mites underlines their clinical importance in the Central European region. It needs to be emphasized that in this paper we focus only on sensitization rates and not their clinical relevance; carrying out the latter analysis without using specific provocation tests might become rather complicated and is not realistic in such large cohorts.

### Cockroach

It has been suggested that a cocktail of five allergens Bla g 1 and/or Per a 1, Bla g 2, Bla g 4, Bla g 5, and Bla g 7 and/or Per a 7 would be expected to diagnose 50–64% of cockroach-allergic patients [15, 16]. The degree of homology between the lipocalin Bla g 4 and the mammalian lipocalins is low, and only small cross-reactivity with these mammalian allergens would be expected [8]. We did not analyze this cross-reactivity because of the very low rate of sensitization to Bla g 4 in our patients. The low rate of sensitization to cockroach-derived molecules was caused probably rather by the low presence of cockroaches in our climate than by missing molecules in the used assay.

### Mites

According to several studies, HDM sensitization and allergy are considered to be the most frequent among inhalant allergies [5, 17, 18]. In our conditions, it holds the fourth position behind grass pollen (sensitization frequency for Phl p 1 is 60.8%), birch pollen (sensitization frequency for Bet v 1 is 47.3%), and cat allergens (sensitization frequency for Fel d 1 is 31.5%) [6], what differs from data coming from other regions [17, 19, 20]. This discrepancy may be caused by geographical differences or may be due to the selected population, mainly consisting of adults with predominant respiratory allergy. Higher sensitization rate to grass pollen in adolescents was also shown in a study from northern Italy while the leading position of mites was pronounced clearly in smaller children [19].

It is known that patients sensitized to mites are not always sensitized to the molecules used in our study, other molecules may also play a role, but the number of patients sensitized only to these other molecules is generally very low [21–25]. Moreover, cysteine protease (Der p 1, Der f 1) signaling has been described to have a strong TH2 up-regulation effect, and group 2 allergens have been shown to bind TLR4 via binding LPS, thus having a stronger immunogenic potential further enhancing the complexity of mite allergy. Hence, these molecules may play a leading role in the atopic march, and a differential sensitization rate in children compared to adults may be present [26]. Sensitization to group 1 allergens was shown to be more frequent in children [18], thereby suggesting a possible role of group 1 allergens in the onset of sensitization, perhaps mediated by their proteolytic activity and direct epithelial damage [27]. The more frequent sensitization to group 2 allergens in the adult population suggested a later sensitization to group 1 allergens what was documented by observation of a birth cohort [26]. The ability of group 2 allergens to bind directly to TLR4 might be an explanation for this observation. These facts may also explain that in the case of mites the higher the exposure, the more severe the clinical allergic condition, in contrast to cat allergens where overexposure seems to lead to tolerance [28].

*Dermatophagoides pteronyssinus* is the most widespread mite all over the world; it predominates especially in humid regions where the climate is more influenced by the ocean. *Dermatophagoides farinae* is supposed to occur more in the continental regions of Europe and the Mediterranean area, but most countries have mixed populations [22, 29]. In our patients, sensitization to *D. pteronyssinus* and *D. farinae* was almost equal in contrast to Spain where the IgE-prevalence to *D. pteronyssinus* allergens was found to be slightly higher than to *D. farinae* allergens [18].

Although the pyroglyphid HDM *D. pteronyssinus* and *D. farinae* seem to predominate, glyciphagid mites may also be important in some regions [18, 21, 29].

*Blomia tropicalis* is the most important house dust mite from the family glycyphagidae. It is most abundant in tropical regions [4, 29]. The major allergen Blo t 5 shows 40% sequence homology with Der p 5, but it was reported not to cross-react with each other [29]. As *B. tropicalis* does not form part of the acarofauna in Central Europe, the not negligible sensitization rate in our patients seems to be attributable rather to potential cross-reactivity among allergens produced by different mite species (mite group 5/21 molecules) than to true sensitization. This assumption is also supported by low levels of sensitization to Blo t 5 in our group. The results of another study suggested that allergens different from those belonging to group 5 may also be responsible for the partial cross-reactivity among different mite species [30].

*Lepidoglyphus destructor*, a glycyphagid storage mite, may also become a HDM [29]. Sensitization from domestic exposure was reported from Sweden and France. It is not possible to decide whether positivity to Lep d 2 in our patients was due to true sensitization or cross-reactivity within mite group 2 molecules. The absence of mono-sensitizations to Lep d 2 (in the frame of mite-derived molecules) in our patients testifies rather for Lep d 2 sensitization due to cross-reactivity with other group 2 allergens, what is in contradiction to the data suggesting that no such cross-reactivity exists [18].

Mite sensitized patients in our group were usually co-sensitized to several mite-specific components; monosensitization was markedly less frequent (Fig. 2). Der f 1 and Der p 1 (cysteine proteases) and Der f 2 and Der p 2 (lipid binding proteins) are assumed to be the specific components most commonly involved in mite allergy. The predominant sensitization, both regarding prevalence and intensity, to mite group 2 molecules was already described [17, 18, 25] and confirmed by our results. In a French study, predominant sensitization to Der p 1 (93%) was detected [31], and we may speculate that this difference might be due to the different population studied. The same French study also detected a higher frequency of sensitization to Der p 10 (28%) signaling the primary sensitizing agent possibly being shrimp which is much more consumed in the Mediterranean area than in Central Europe.

Tropomyosins are molecules responsible for cross-reactivity among mites, shrimp, and cockroach [32]. IgE binding to the mite group 10 allergens is rare in Europe [21] and Australia [33] and, from one study, in US subjects allergic to both HDM and cockroach [34]. Similarly, in this study’s population group, sensitization to individual tropomyosins was considerably less frequent than sensitization to more tropomyosin molecules together suggesting present cross-reactivity in the frame of this group of molecules (Fig. 3).

Sensitization to the tropomyosin Der p 10 was not observed frequently (in 1.9%) in our patients, and co-sensitizations with other mite-specific molecules were not regular (in 0.8%) suggesting the possible different route of sensitization in a considerable proportion of the patients. Tropomyosins represent clinically relevant seafood allergens, but the role of mite tropomyosin, Der p 10, in house dust mite allergy has not been studied in detail. A hypothesis that tropomyosin sensitization may indicate a true food allergy independent of mite respiratory disease has been proposed [18]. Another hypothesis pretends that Der p 10 may be a diagnostic marker for mite-allergic patients with additional sensitization to allergens other than Der p 1 and Der p 2. Such patients may require attention when allergen-specific immunotherapy is considered [35].

The high rate of simultaneous sensitizations to different mite components (Fig. 2) may be explained either by co-sensitizations or by cross-reactivities. Specific IgE to Der p 2/Der f 2 is almost fully cross-reactive, but no cross-reactivity was described with Lep d 2 [18]. Group 1 allergens are also cross-reactive, but in some patients, a species-specific response was observed [18]. Relatively high rate of sensitization both to group 1 and 2 allergens in our patients indicates the greater role of co-sensitizations.

## Conclusions

The vast majority of mite-sensitized patients in our group showed polysensitization to two or more components derived from both major HDM, i.e., *D. pteronyssinus* and *D. farinae*. On the contrary, only a minority was sensitized to mite-derived tropomyosin, and these sensitizations were not frequently connected to sensitization to other mite-derived molecules, suggesting dominating different route of sensitization via food-derived tropomyosins.

It is necessary to stress the importance of the knowledge of allergen content in mite extracts used for diagnostic and therapeutic purposes as a practical implication from this study. Several studies have focused on this issue and analyzed the composition of several commercially available preparations concerning their qualitative and quantitative allergen composition. There are evident considerable differences in the allergen content among the commercially available extracts [9–11]. Although some authors question the necessity of tailoring of allergen immunotherapy to the sensitizing species [22], we consider this to be important for the optimal efficacy of this treatment. Group 2 allergens are highly cross-reactive, but as group 1 sensitization could be species specific in some patients and its prevalence is higher in children, an adequate balance of major mite species and major allergens must be considered in the design of mite allergy vaccines. Regarding the sensitization patterns of patients within the Central European region, it is necessary to point out the importance of quantifying at least three major mite components Der f 1, Der p 1 and Der f 2 (or Der p 2). Such information is crucial for effective diagnosis and treatment. Besides these molecules, Der p 23, a new major house dust mite allergen, should be considered to be an important component for allergen-specific immunotherapy as well [26, 36, 37]. The importance of eventual further allergens (so-called “mid-tier” allergens—e.g., Der p 5) in this context has yet to be elucidated. There is an urgent need for rigorous, long-term clinical trials with an efficacy criterion to find the consensus on the dose of individual molecules in HDM-allergen-specific immunotherapy [37, 38].

## Abbreviations

HDM: house dust mite
IgE: immunoglobulin E
ISAC: immuno solid-phase allergen chip
STROBE: strengthening the reporting of observational studies in epidemiology
LPS: lipopolysaccharide
